# Crotoxin promotes macrophage reprogramming towards an antiangiogenic phenotype

**DOI:** 10.1038/s41598-019-40903-0

**Published:** 2019-03-12

**Authors:** Luciana de Araújo Pimenta, Maíra Estanislau S. de Almeida, Marisa Langeani Bretones, Maria Cristina Cirillo, Rui Curi, Sandra Coccuzzo Sampaio

**Affiliations:** 1 Laboratory of Pathophysiology, Butantan Institute, Av. Vital Brazil, 1500, 05503-900 São Paulo, SP Brazil; 2 Department of Pharmacology, University of São Paulo, Av. Prof. Lineu Prestes, 1524, 05508-900 São Paulo, SP Brazil; 3 Department of Cell and Developmental Biology, University of São Paulo, Av. Prof. Lineu Prestes, 1524, 05508-900 São Paulo, SP Brazil; 4 Department of Physiology and Biophysics, Institute of Biomedical Sciences, University of São Paulo, Av. Prof. Lineu Prestes, 1524, 05508-900 São Paulo, SP Brazil; 5 Interdisciplinar Post-Graduate Program in Health Sciences, Cruzeiro do Sul University, 868 Galvão Bueno, 01506-000 São Paulo, Brazil

## Abstract

Crotoxin (CTX) is the primary toxin of South American rattlesnake *Crotalus durissus terrificus* venom. CTX reduces tumour mass, and tumour cell proliferation and these effects seem to involve the formation of new vessels. Angiogenesis has a key role in tumour growth and progression and is regulated by macrophage secretory activity. Herein, the effect of CTX on macrophage secretory activity associated with angiogenesis was investigated *in vitro*. Thymic endothelial cells (EC) were incubated in the presence of macrophages treated with CTX (12.5 nM) or supernatants of CTX-treated macrophages and endothelial cell proliferation, migration and adhesion activities, and the capillary-like tube formation in the matrigel-3D matrix was measured. Angiogenic mediators (MMP-2, VEGF and TNF-α) were measured in the cell culture medium. Macrophages pre-treated with CTX and supernatant of CTX-treated macrophages inhibited EC proliferation, adhesion to its natural ligands, and migration (as evaluated in a wound-healing model and Time Lapse assay) activities. Decreased capillary-like tube formation and MMP-2, VEGF and TNF-α levels in the supernatant of macrophages treated with CTX was also described. CTX promotes macrophage reprogramming towards an antiangiogenic phenotype.

## Introduction

Crotoxin (CTX) is a β-heterodimeric neurotoxin formed by the noncovalent association of two subunits, one acidic termed crotapotin (subunit CA~9.5 kDa) and one basic (subunit CB~14.5 kDa) known as phospholipase A_2_ (PLA_2_)^1^. CTX corresponds to 60% of the total venom of the South American rattlesnake *Crotalus durissus terrificus* and its molecular weight is 24–26 kDa, isoelectric point of 4.7, and exhibits phospholipase activity, neurotoxic (blockage of neuromuscular transmission) and myotoxic^2–4^ properties. Sixteen isoforms of CTX were identified as a result of a random combination of four CA isoforms (CA1, CA2, CA3 and CA4) and four isoforms of CB (CBa_2_, CBb, CBc and CBd)^5^. The combinations of these isoforms determine the formation of different complexes, responsible for the different pharmacological and biological properties reported for CTX^6^.

Anti-inflammatory, antitumour and immunomodulatory properties of CTX have been disclosed either in humans (antitumour effect) or experimental animal models^7–9^, for review^10–14^. CTX is nephrotoxic and has potent effects on neuromuscular activity and cardiovascular system function^9^, for review. CTX raises glucose and glutamine utilization and oxidation inhibits spreading and phagocytosis activities^15^ and increases production of hydrogen peroxide and nitric oxide by macrophages^10^. In this sense, it is important to point out the immunomodulatory effects of CTX, accompanied by tumor regression, observed *in vivo* experimental models, occurs after administration of low concentration (μg), with rapid onset and long duration and are observed for up to 14 days after a single dose^10^. After this period no manifestation of neurotoxic, nephrotoxic, myotoxic actions are observed. Associated with this fact, mice injected daily with progressively increasing doses of CTX develop tolerance to the lethal action of the toxin. The treated mice tolerated daily doses of CTX 20 to 35 times greater than the original LD50, without the characteristic signs of toxicity. In addition, clinical studies have demonstrated that administration of CTX has been conditioned by the absence of dose-limiting toxicity from the previous dose administered, along with pain relief related to pancreatic cancer and arthritis (Public Patent US 2013/0129706 A1). Macrophages pre-incubated with CTX and co-cultured with LLC WRC 256 tumour cells exhibit increased production of reactive oxygen and nitrogen species and secretion of IL-1β and lipid mediators as lipoxin A_4_ (LXA_4_) and its stable analogue 15-epi-LXA_4_. The secretory activity of macrophages has been associated with inhibition of tumour cell proliferation^16^. We previously reported a marked reduction in the growth of solid tumours in the flank and paw of rats by 88% and 40% respectively^10,14,17^. This action was accompanied by both a decrease in the formation of new vessels and vessel thickness, suggesting that CTX inhibition of tumour growth compromises the events of angiogenesis^14^. To understand how CTX interferes with the tumor microenvironment *in vivo*, a first *in vitro* study carried out by our group demonstrated the direct antiangiogenic activity induced by CTX on the key events involved with angiogenesis process, responsible for adhesion and migration functions, such as protrusion formation of actin cytoskeleton of the thymic endothelial cells^18,19^. Furthermore, there is evidence that increased levels of LXA_4_ and its analogue 15-epi-LXA_4_ possibly secreted by macrophages are involved in the antitumor and antiangiogenic actions of CTX^14^. In spite of this information, the involvement of macrophages in the antiangiogenic activity of CTX remains covered.

Macrophages play essential roles in the innate and adaptive immune responses^20^, for review. These cells secrete a large number of mediators with several and sometimes inverse functions^20^, for review. Macrophages play a crucial role in the initiation and promotion of tumorigenesis and angiogenesis^21,22^, for review^23–27^ and may comprise up to 80% of the cell mass in the solid tumour^28,29^. These cells can promptly reprogram metabolism and function towards a pro-inflammatory (M1) or anti-inflammatory (M2) phenotype and secretion of pro- and anti-antiangiogenic mediators^20^, for review. Macrophages promote neovascularization through secretion of proangiogenic factors such as tumour necrosis factor-α (TNF-α) and endothelial growth factors (VEGF)^20^, for review^30–33^. The VEGF family is the most potent inducer of angiogenesis and lymphangiogenesis^34,35^. TNF-α is one of the tumor-associated cytokines with angiogenesis properties^33,36,37^. Macrophages also release metalloproteases (MMPs) that degrade the extracellular matrix and favor tumour angiogenesis. The primary MMPs secreted by macrophages are MMP-9 and MMP-2^38,39^, for review. As mentioned above, macrophages secrete LXA_4_ and its stable analogue (15-epi-LXA_4_) with antiangiogenesis properties. These lipid mediators are generated through lipoxygenase and exert specific biological effects upon binding to membrane G-protein coupled formyl peptide receptors-FPRs (also known as ALXR) that have been reported in several cell types including macrophages^40,41^. These mediators have inhibitory effects on tumour growth^42^ and endothelial cell proliferation^26^ and suppress production of angiogenic growth factors^25,26,43^. Macrophages secrete both angiogenic and antiangiogenic factors and so play a central role in the tumour and inflammatory induced-neovascularization^26,44^, for review.

The effect of CTX on rat macrophage secretory activity associated with angiogenesis was investigated in thymic endothelial cells (EC) in culture. EC was incubated in the presence of macrophages treated with CTX or supernatants of CTX-treated macrophages. The following measurements were performed: endothelial cell proliferation, migration and adhesion activities, capillary-like tube formation in a matrigel-3D matrix, and production of angiogenic mediators (MMP-2, VEGF and TNF-α). Also, we performed the measurements and tube-forming assay by human endothelial cells (HUVEC-CS) on the matrigel-3D matrix using the supernatant of human macrophages (THP-1 macrophage differentiated) pretreated with CTX.

## Results

### Macrophages treated with CTX decreased the proliferative capacity of endothelial cells

Initially, two concentrations of CTX were tested (12.5 nM and 50 nM). Similar inhibitory effects were observed, and so the lower concentration was chosen to perform the study (see Supplementary Fig. S1). At this concentration, CTX did not cause cell death as assessed by trypan blue dye and the crystal violet assays and reported in our previous study^16^.

**Figure 1:**
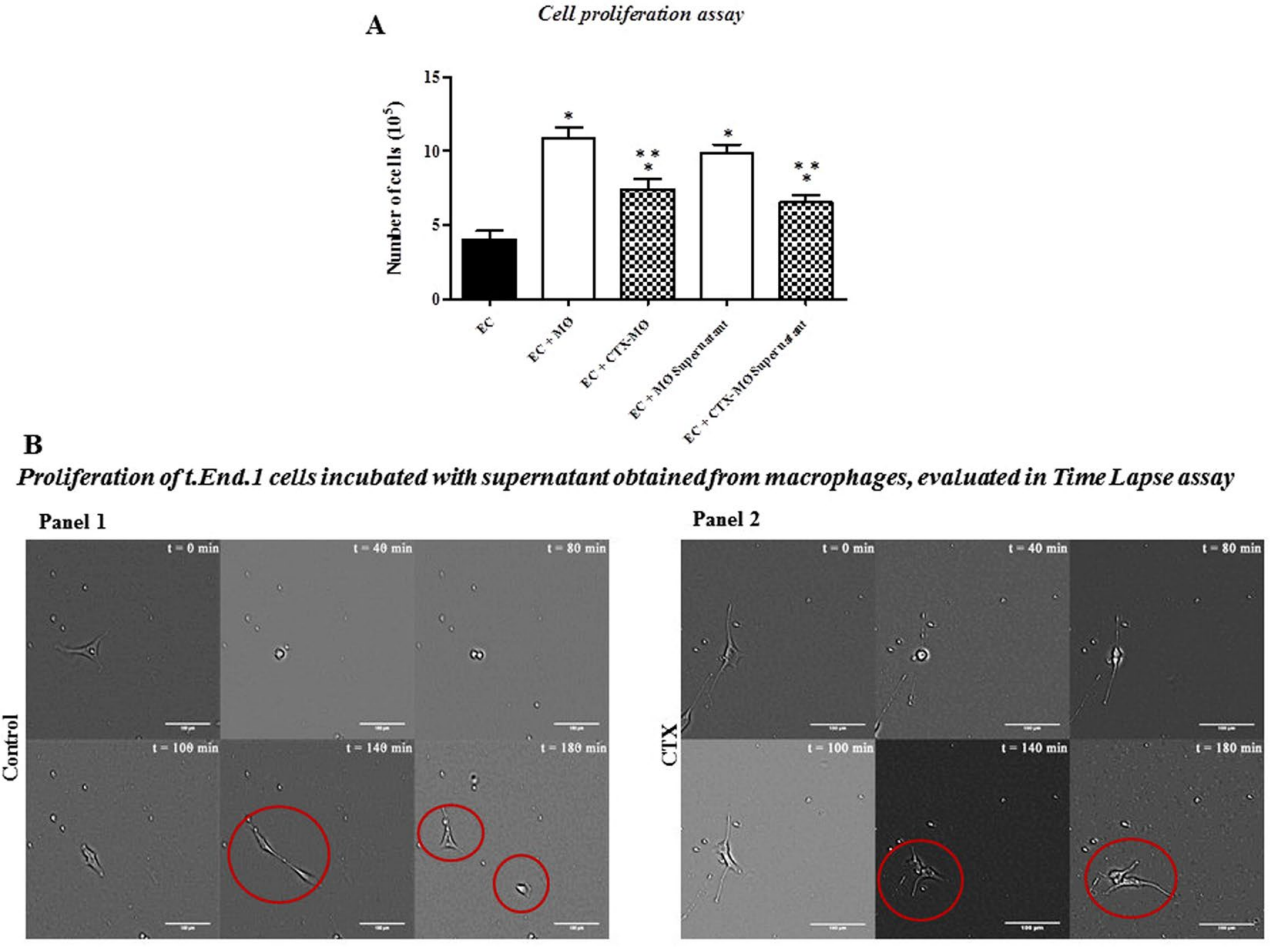
Effect of co-cultures of macrophages pretreated with CTX on the EC proliferation. EC (5 × 10^5^ cells/well in 24-well plates) were incubated for 24 hours at 37 °C, and 5% CO_2_ in the presence of CTX treated or untreated macrophages (1 × 10^6^/well, previously incubated with 12.5 nM of CTX, for 2 h). EC was also incubated in culture medium containing supernatants of CTX treated or untreated macrophages under similar conditions. For details of the experiments, please see the Materials and Methods section. The results are expressed as the number of cells and presented as mean ± s.e.m. of six samples per group of three distinct assays. In (**A**) *p < 0.05, compared to the group incubated with EC (control) and **p < 0.05, significantly different from mean values for groups to their respective controls (t.End.1 + Untreated Mϕ or t.End.1 + Untreated Mϕ supernatant). The images in the panel (B) were obtained in the 5x objective, after 180 min of the migration cell evaluated in Time Lapse assay, in the presence of supernatants of macrophages controls or CTX-treated. °Cytoskeleton cells during division cell.

We investigated whether the inhibitory activity of macrophages treated with CTX would be dependent on cell-cell contact or the supernatant of cultured macrophages could have the same effect. To this end, EC was incubated for 24 hours in the presence of supernatants from macrophage monocultures pre-treated with CTX (12.5 nM). The proliferative capacity was significantly decreased (by 34%) as compared to EC incubated with supernatants from untreated macrophage (Fig. 1A). The co-cultures of macrophage and EC (cell-cell) were performed concomitantly to compare the magnitude of the responses. CTX pre-treated co-cultured macrophages inhibited EC proliferation (by 32%) as compared with EC co-cultured untreated macrophages (Fig. 1A). So, similar CTX inhibition magnitude was observed in the two protocols.

Cytoskeletal dynamics of EC during proliferation assay was evaluated in type I collagen-coated wells in the presence of supernatant of untreated macrophages (Fig. 1B, Panel 1). After 40 min of the incubation, the cytoskeleton of the EC became retracted, and mitosis was observed after 80 min. Between 100 min and 140 min, spreading cells on collagen coating was still seen. Separation of the cells to continued migration appeared after 180 min run. EC incubated in the presence of CTX-treated macrophage supernatants (Fig. 1B, Panel 2) had poor cytoskeleton separation between 140 min and 180 min run, indicating alteration of cytoskeletal dynamics that impaired EC proliferation.

### Macrophages treated with CTX inhibit the endothelial cell migration on type I collagen

**Figure 2:**
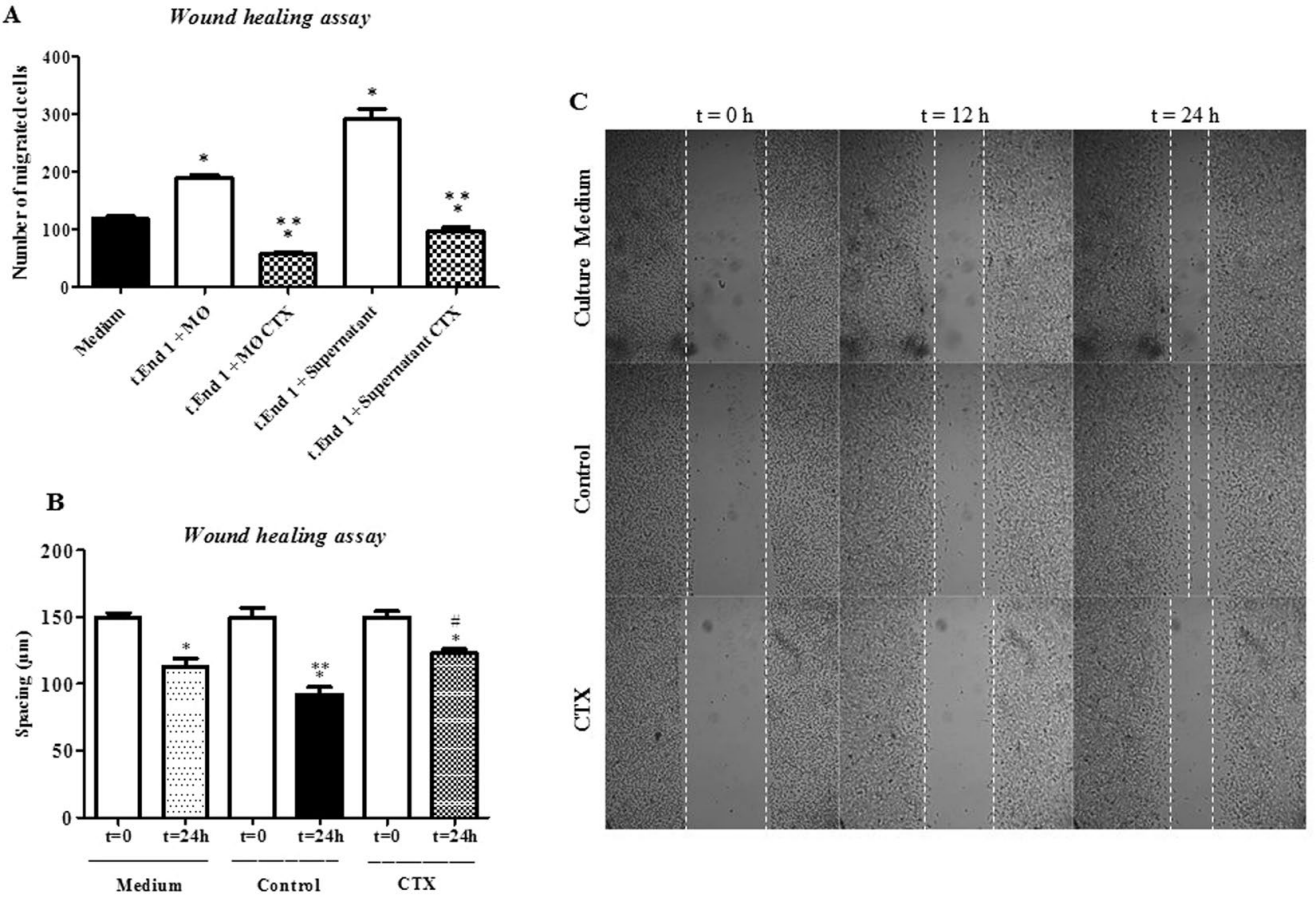
Effect of co-culture of macrophages pre-treated with CTX and the supernatant from these cells on EC migration evaluated in time-lapse assay. EC was incubated in the presence of macrophages treated or not with CTX (12.5 nM) or with supernatants from these macrophages. Then, the plate was coupled to the equipment InCell Analyzer 2200 GE in the 10x objective, for 24 h. In (**A**), the results are expressed as the number of migrated cells in the empty field. *p < 0.05, significantly different from mean values for group incubated with t.End. 1 (control) and **p < 0.05, significantly different from mean values for groups to their respective controls. In (**B**), the spacing was calculated from T0 (initial incubation up to 24 h). *p < 0.05 compared to T0, the respective groups. **p < 0.05 significantly different from mean values for culture medium and ^#^p < 0.05 significantly different from mean values for Untreated MØ group. These parameters were assessed using ImageJ software Data are the average of five samples of each group ± S.E.M and represent two distinct trials. (**C**) is representative of response-time images obtained in real time from T0 (start of culture) and after 12 h and 24 h of the EC incubation only in culture medium (Medium), supernatant from Untreated macrophages (Control) or supernatants from CTX-treated macrophage, showing the spacing at T0 (initial time) and T24 (end time analysis).

EC incubated with macrophages pre-treated with CTX or supernatant obtained from the cultured cells under this condition had a significant decrease (by 48%) of migration activity as compared with cells cultured with untreated macrophages (Fig. 2A). After 24 hours in culture, a significant decrease of the spacing between the edges of the field compared to the respective T0 was observed: by 24% for EC incubated with culture medium and by 38% for EC incubated with supernatant of untreated macrophages. The fields related to EC incubated for 24 h in the presence of supernatant of macrophages treated with CTX exhibited spacing (by 18%), demonstrating inhibition of EC migration to the empty field (Fig. 2B). Figure 2C is representative of each analyzed culture. Two-dimensional (2D) migration was evaluated through time-lapse assay and a decrease (15%) in migration velocity of the EC incubated in the presence of the supernatants of macrophages pre-treated with CTX was found: Control- macrophage supernatants (mean: 71.68 ± Std error: 3.56, N = 41 and CTX-treated macrophage supernatants (mean: 60.75 ± Std error: 3.39, N = 36, p < 0.0152) [see Supplementary Videos S1 and S2 (Wound Healing in Time-Lapse assay)]. Even after 18 h post-migration, EC exhibited altered migratory behavior and altered the cytoskeletal dynamics and the formation of lamellipodia-like structures in EC, and consequently the migration of these cells [see Supplementary Videos S3 and S4 (Migration in Time-Lapse assay)]. From these images the cell velocity and was measured [see Supplementary Data (Cell velocity numerical data)].

### Macrophages treated with CTX inhibit endothelial cell adhesion to matrix components

**Figure 3:**
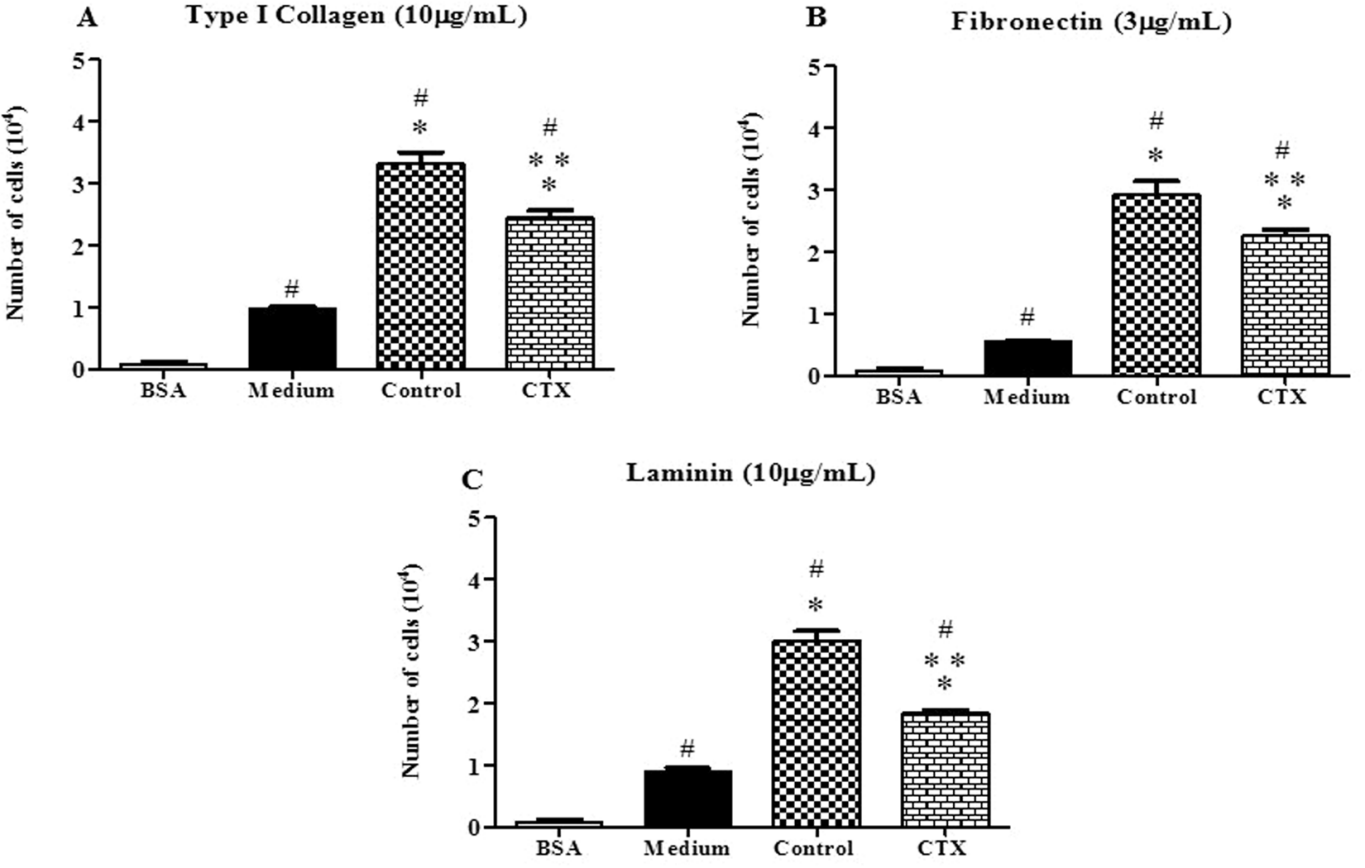
Effect of supernatants from macrophages pretreated with CTX on EC adhesion to different components of the extracellular matrix. T.End.1 cells were incubated for 24 h in the absence (only RPMI 1640) or in the presence of macrophage supernatants of treated or not with CTX (12.5 nM). After this period, the cells were washed, suspended and cell suspension (1 × 105/100 µL/well) was added to Maxsorp plates (Nunc®) containing 96 wells, previously sensitized with the different components of matrix: (**A**) type I collagen – 10 µg/well; (**B**) fibronectin – 3 μg/well and (**C**) laminin-10 µg/well. After 1 h, the adhered cells were evaluated by MTT assay. The experiments were conducted in octuplicates. The values obtained were entered into GraphPad INSTAT program; Software V2.01 for conversion of optical density (OD) in the number of adhered cells. *p < 0.001, compared to the control group (culture medium only). **p < 0.001, significantly different from mean values for supernatant of macrophage control group. ^#^p < 0.001, significantly different from mean values for the BSA group.

Supernatants from untreated cultured macrophages increased the adhesion of EC to the matrix components (by 3.35-fold for Type I Collagen; 5.28-fold for fibronectin and 3.36-fold for laminin) when compared to EC incubated with RPMI-1640 culture medium only (Fig. 3). In contrast, the supernatant of CTX-treated macrophages inhibited (by 26%) the adhesion of EC to type I collagen (Fig. 3A). Inhibition was also observed for fibronectin and laminin by 23% and 39%, respectively, under this latter condition (Fig. 3B,C).

### Macrophages treated with CTX inhibit the capillary-like tube formation in the matrigel-3D matrix

**Figure 4:**
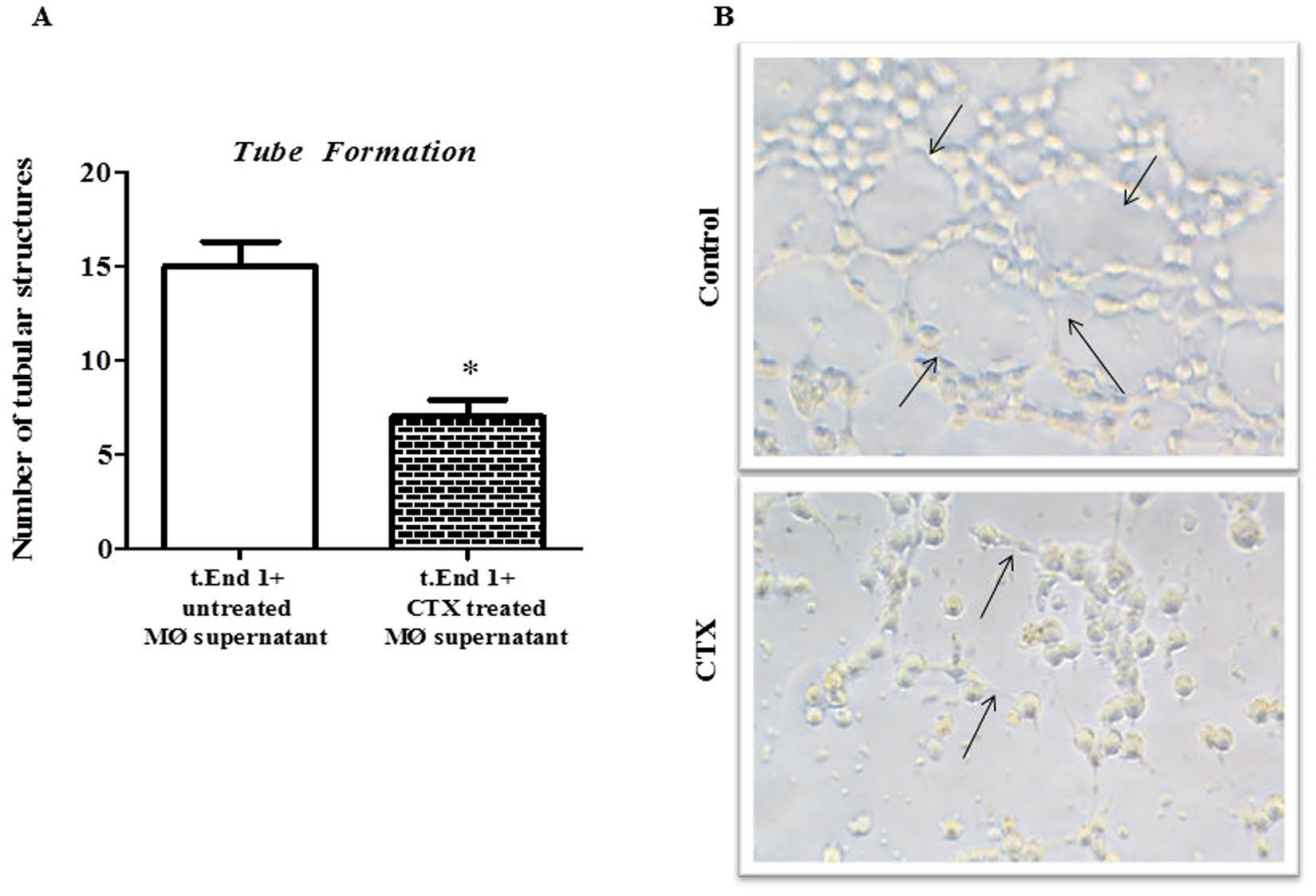
Effect of supernatants from macrophages pre-treated with CTX on the formation of tubule-like structures in 3D-matrigel. EC cells (2.5 × 10^4^) were suspended in 50 µL RPMI-1640 culture medium or 50 µL of supernatants obtained from macrophage monolayer treated or not with CTX (12.5 nM). The cells were then added on matrigel and incubated for 2 h at 37 °C and 5% CO_2_. In (**A**), the results are expressed in the number of tubular structures 5 and represent the field count of three different samples of each group. *p < 0.05, significantly different from mean values for control group only incubated with RPMI 1640. (**B**) Represents the tube formation of EC incubated with the supernatant of macrophages incubated with culture medium (Control) and with the supernatant of macrophages pretreated with CTX. Images obtained at the 40x objective. Connecting structures.

After 2 hours of incubation in the presence of untreated macrophage supernatant, EC had the extensive formation of capillary-like structures with elongated and thin structures allowing the cell-to-cell contact (Fig. 4). EC incubated in the presence of supernatants from CTX- pre-treated macrophages had a significant reduction in the capillary forming features (by 53%) with few connecting structures when compared with untreated macrophage supernatant.

### CTX decreases angiogenic mediators release by macrophages

**Figure 5:**
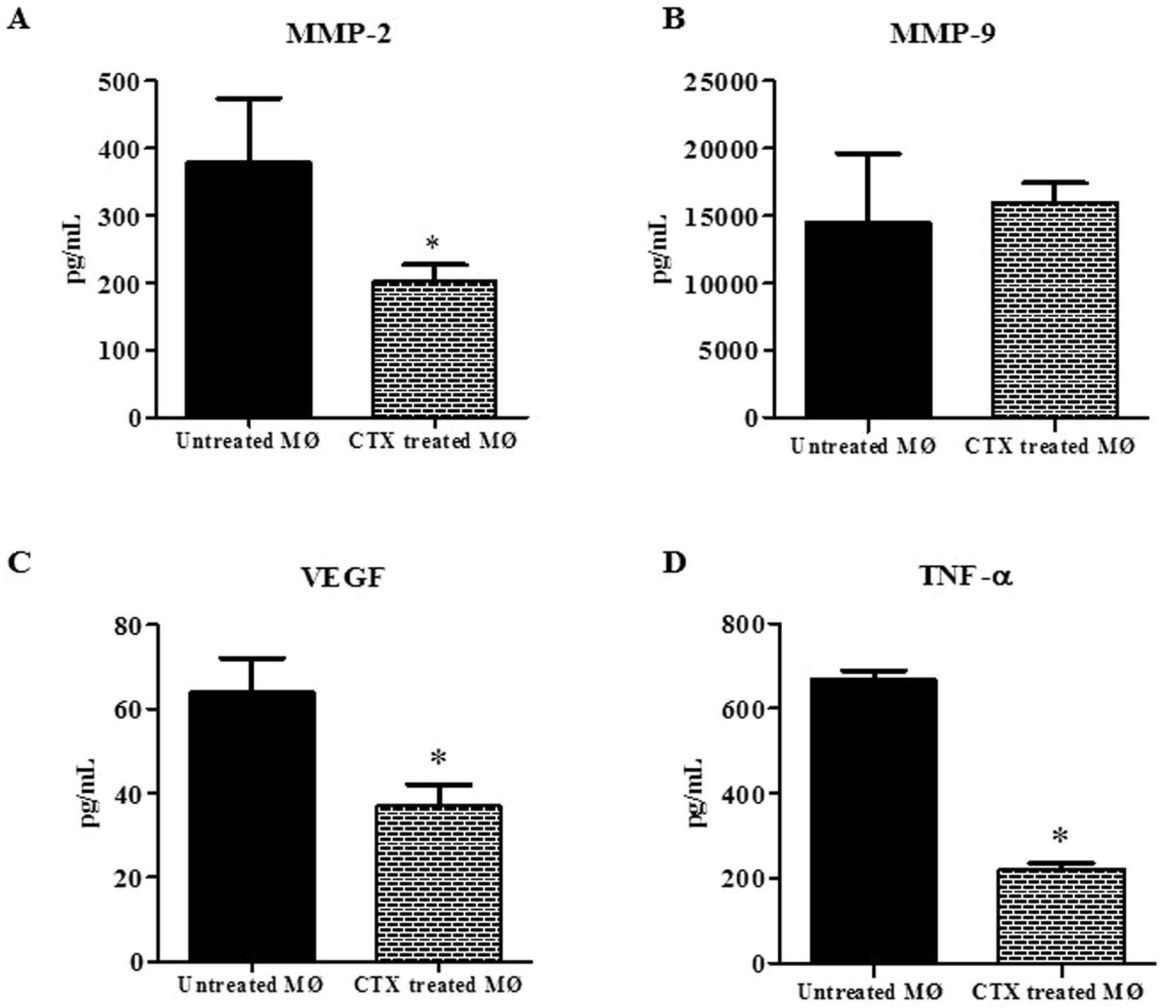
Effect of CTX on MMP-2, MMP-9, VEGF and TNF-α release by macrophages. Macrophages (1 × 10^6^/mL) were adhered for 1 hour and incubated in the absence or presence of CTX (12.5 nM) for 2 hours at 37 °C and 5% CO_2_. After this period, the plates were washed and incubated for 24 h in the presence of the fresh medium. Then, the supernatants were collected for determination of the concentration of MMP-2, MMP-9, VEGF and TNF-α by means of enzyme immunoassay (EIA) using a commercial kit. Data are mean ± S.E.M. Data are the average of four samples of each group ± S.E.M and represent two distinct trials. In (**A,B**) *p < 0.0001, significantly different from mean values for control group. In (**C**) *p < 0.01, compared with the respective control group.

CTX decreased (by 47%) the secretion of MMP-2 by macrophages as compared to respective controls (Fig. 5A). CTX also reduced VEGF (by 42%) and TNF-α (by 67%) secretion by macrophages (Fig. 5C,D, respectively). Regarding the secretion of MMP-9, no difference was observed between CTX-treated and untreated macrophages (Fig. 5B). These results confirm the inhibitory action of CTX on the secretion of angiogenic mediators by macrophages and might be associated with the reduction of vessel formation in a tumour environment.

### Formyl peptide receptor (FPR), LXA4 and 15-Epi-LXA4 binding receptors, are involved in the inhibitory action of CTX-treated macrophage supernatants on endothelial cell function

**Figure 6:**
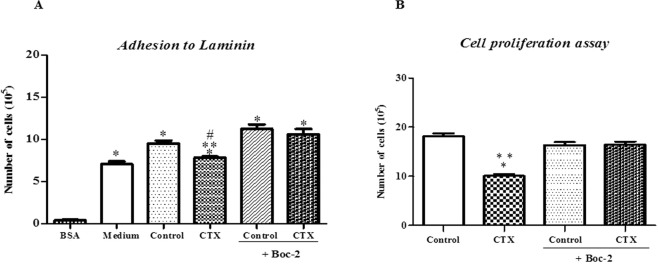
Effect of Boc-2 on inhibitory action of macrophages pretreated with CTX on EC proliferation and adhesion. Macrophages (1 × 10^6^) were incubated with Boc-2 (100 µM), for 15 minutes at 37 °C. After that, the macrophages were washed with PBS and incubated with CTX (12.5 nM) for 2 h. After this period, they were incubated in fresh medium for 24 h. In (**A**), the number of cells in a Neubauer chamber under light microscopy. In **A** Data are mean ± S.E.M. *p < 0.05 compared with the control group (N = 6) and CTX (N = 6). **p < 0.05 compared with control + Boc-2 and CTX + Boc-2 groups (n = 6 each). In (**B**), cell adhesion capacity was determined by MTT assay. The experiments were conducted in octuplicates. The values obtained were entered into GraphPad INSTAT program; Software V2.01 for conversion of optical density (OD) in the number of adhered cells. *p < 0.001, compared to BSA group. **p < 0.001, significantly different from mean values for control group. ^#^p < 0.001, significantly different from mean values for control + Boc-2 and CTX + Boc-2 groups. These graphs are representative of two different experimental trials for each model.

Supernatants from macrophages preincubated with Boc-2 (100 µM, for 15 min) and treated with CTX (12.5 nM) did not exhibit the inhibitory action on EC adhesion (Fig. 6A) and proliferation (Fig. 6B) as compared with their respective controls incubated in culture medium only or in the presence of Boc-2 + culture medium. These results highlight the importance of FPRs for the CTX actions on the function and secretory activity of macrophages.

### CTX-treated human cell-supernatants inhibit the proliferation, adhesion and migration capacities by the HUVEC

**Figure 7:**
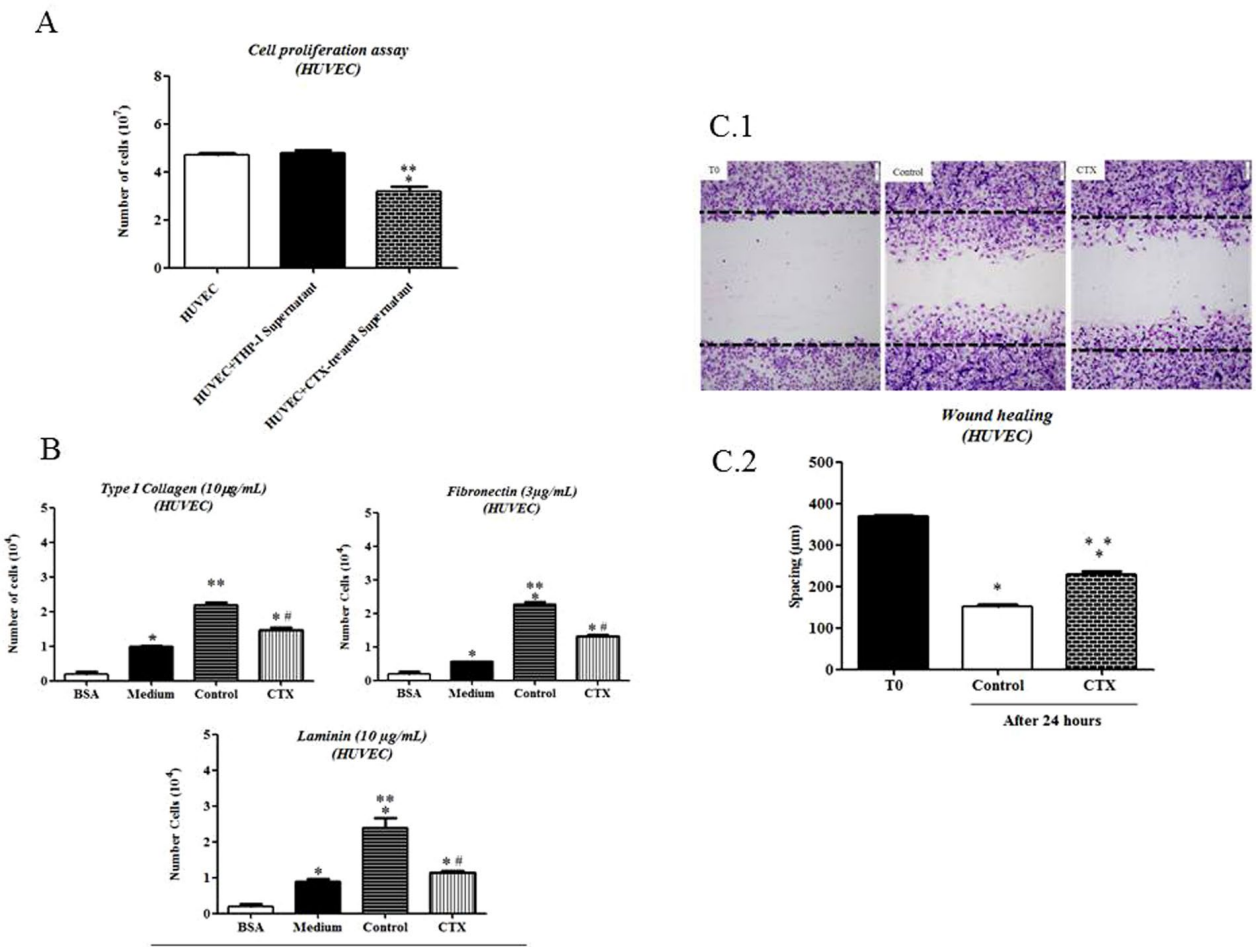
Effect of supernatants from Human lineage cells pretreated with CTX on the HUVEC function. In (**A**), THP-1- differentiated macrophages were incubated or not in the presence of CTX treated (12.5 nM) for 2 hours, washed and incubated with fresh medium for 24 h at 37 °C and 5% CO_2_. HUVEC-CS was incubated in culture medium containing supernatants of CTX treated or untreated THP-1-macrophage differentiated under similar conditions. Number of cells was determined in a Neubauer chamber under light microscopy. Data are mean ± S.E.M. *p < 0.05 compared with the control group (N = 5) and CTX (N = 5). In (**B**), cell adhesion capacity was determined by MTT assay. The experiments were conducted in octuplicates. The values obtained were entered into GraphPad INSTAT program; Software V2.01 for conversion of optical density (OD) in the number of adhered cells. Data are mean ± S.E.M. *p < 0.001, significantly different from mean values for the BSA group. ** p < 0.001, compared to the control group (culture medium only, N = 5). ^#^p < 0.001, significantly different from mean values for supernatant of macrophages from control group (N = 5). (**C.1**) is representative of response-time images obtained from T0 (start of culture) and after 24 h of the HUVEC-CS incubation in supernatant from Untreated THP-1- differentiated macrophage (Control) or supernatants from CTX-treated THP-1- differentiated macrophage, showing the spacing at T0 (initial time) and T24 (end time analysis). In (**C.2**), HUVEC-CS migration capacity was evaluated from the spacing, calculated from T0 (initial incubation up to 24 h). Data are mean ± S.E.M and represent two distinct trials. *p < 0.001, compared to the T0 group. **p < 0.001, significantly different from mean values for supernatant of THP-1- differentiated macrophages from control group (N = 6). These parameters were assessed using ImageJ software.

We also investigated whether human macrophages would be susceptible to the inhibitory effect of CTX. For this, we performed experimental assays using differentiated macrophages from THP-1. In the same way, the human endothelial cell line (HUVEC) was used to evaluate the immunomodulation activity of THP-1-differentiated macrophages on the different function measurements. To this end, HUVEC was incubated for 24 hours in the presence of supernatants from THP-1- differentiated macrophage pre-treated with CTX (12.5 nM). The proliferative capacity of HUVECs was significantly decreased (by 33%) compared to HUVEC with supernatants from untreated THP-1- differentiated macrophage (Fig. 7A).

Similarly to that observed in the assays using macrophages obtained from rat peritoneum and murine endothelial cell line (t.End.1), the supernatants from untreated THP-1- differentiated macrophage increased the adhesion of HUVEC to the matrix components (by 4.2-fold for Type I Collagen; 1.92-fold for fibronectin and 3.72-fold for laminin) when compared to HUVEC incubated with RPMI-1640 culture medium only (Fig. 7B). Already, the CTX-treated human macrophages-supernatants inhibited the adhesion of HUVEC to type I collagen, fibronectin and laminin by 33%, 42% and 52%, respectively (Fig. 7B).

The migration assay using human macrophages demonstrates after 24 hours in culture, a significant decrease (58%) of the spacing between the edges of the field compared to the respective T0 was observed for HUVEC incubated with supernatant of untreated macrophages. The fields related to HUVEC incubated for 24 h in the presence of supernatant of THP-1- differentiated macrophage treated with CTX (12.5 nM) exhibited greater spacing between the edges (by 51%) as compared with cells cultured with untreated THP-1- differentiated macrophage, demonstrating inhibition of HUVEC migration to the empty field (Fig. 7C.2). Figure 7C.1 is representative of each analyzed culture.

### CTX-treated human cell-supernatants inhibit the capillary-like tube formation by the human endothelial cell in the matrigel-3D matrix with the involvement of the formyl peptide receptors

**Figure 8:**
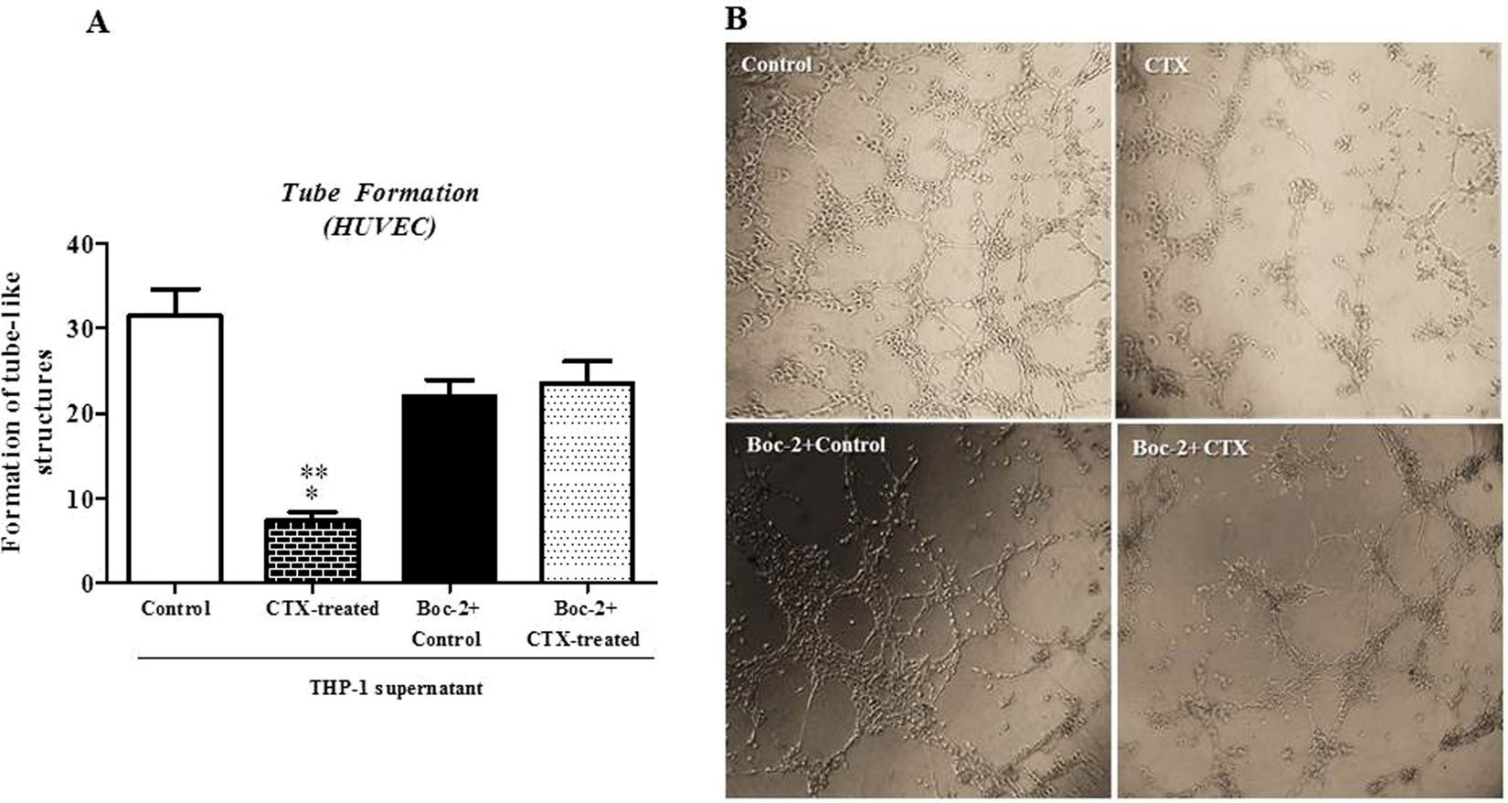
Effect of supernatants from Human lineage cells pretreated with CTX on the formation of tubule-like structures in 3D matrigel and involvement of the FPRs. THP-1-macrophage differentiated was then previously incubated with Boc-2 (100 µM, for 30 min) and then was treated or not with CTX (12.5 nM) for 2 hours, washed and incubated with fresh medium for 24 hours. After a period, HUVEC-CS (2.5 × 10^4^ cell/50 µL) was added 50 µL of the supernatant of differentiated macrophages from THP-1 cells. Then, the total volume of 100 µL was plated on the polymerized Matrigel and incubated for 24 hours at 37 °C, 5% CO_2_. In (**A**), the results are expressed in number tube-like structures and represent the field count of six different samples of each group. *p < 0.0152, significantly different from mean values for control group. **p < 0.0152, significantly different from mean values for Boc-2 + control and Boc-2 + CTX groups. (**B**) Represents the tube formation of EC incubated with the supernatant from differentiated macrophages from THP-1 cells pretreated with medium (Control), with the supernatant from differentiated macrophages from THP-1 cells pretreated with CTX, with Boc-2 + THP-1 cells pretreated with medium (Control) and Boc-2 + supernatant of THP-1 pretreated with CTX. Images obtained at the 40x objective.

The supernatant of human macrophages (THP-1-macrophage differentiated) treated with CTX (12.5 nM) inhibited significantly (77%) the capillary-like tube formation by HUVEC, as compared with HUVEC incubated in the presence of the culture medium only (control), as shown Fig. 8. Supernatants from macrophages preincubated with Boc-2 (100 µM, for 30 min) and treated with CTX (12.5 nM) did not exhibit the inhibitory effect on tube formation by HUVEC cells (Fig. 8) as compared with their respective controls incubated in culture medium only or the presence of the Boc-2 + culture medium. These results reinforce the importance of FPRs for the CTX actions on secretory activity of macrophages, evidencing for the first time, the immunomodulatory effect of the CTX on human cells.

## Discussion

Several pieces of evidence suggest that macrophages act as critical effectors, provoking a pro-angiogenic (M2) outcome during the “angiogenic switch” and play a crucial role in stimulating tumour angiogenesis and progression^45,46^, for review. Studies involving the metabolism of macrophages emphasized the vital link between the metabolic state and the phenotype (M1 and M2) of these cells^47^. In this context, Faiad and colleagues have demonstrated the importance of CTX action on macrophages plasticity to control some pathophysiological processes, involving a stimulatory action CTX on glucose and glutamine metabolism and secretory activity of peritoneal macrophages followed significant inhibition of the subcutaneous development of Walker 256 tumour^10^. Herein, the effects of CTX pretreated macrophage or supernatant obtained from macrophage monocultures pretreated on endothelial cell were *in vitro* investigated. We observed that both macrophages treated with CTX and supernatant from these monocultures inhibited the proliferation capacity and migratory ability of EC after 24 hours of incubation. Besides, the supernatants from monoculture macrophages pretreated with CTX inhibited endothelial cell adhesion to all substrates evaluated (type I collagen, fibronectin and laminin) and this inhibition was more pronounced on laminin. Considering the importance of the proliferation, migration and adhesion events to the formation of new blood vessels, in the present study investigated whether the inhibitory effect of CTX on these developments would compromise the vessel formation. We use Matrigel, a reconstituted basement membrane which can provide similar conditions to that observed in the tumour microenvironment thus offers rapid formation of tubular structures in a 3D system, one simple and easily quantifiable^48,49^. We observe that the formation of capillary-like tube formation on 3D-matrix was also altered in the presence of supernatants from CTX-treated macrophage since it was observed the change of the EC morphology, which prevents the formation of the capillary network in matrigel. It is important to note that the same assay was performed with human cells (HUVEC-CS incubated with CTX-treated supernatant of THP-1 differentiated macrophages). The results show a significant inhibitory effect of this supernatant on HUVEC function and tube formation by HUVEC cells in Matrigel.

These events can be modulated by macrophages, which depending on its activation state secrete substances, which inhibit (M1) or promote (M2) neovascularization^22,46^, for review. Our results show that supernatants from macrophages treated with CTX inhibit all functions of the presently evaluated EC, suggesting that CTX alters the release of mediators secreted by macrophages affecting events involved in angiogenesis. Studies have demonstrated, *in vitro* models, that peritoneal macrophages secrete mediators that modulate the EC proliferation such as cytokines, growth factors, lipid mediators and MMPs^16,27,50,51^. Accordingly, to characterize the inhibitory effect of CTX action on the secretion of these mediators by macrophages was determined by the release of VEGF, TNF-α, and MMP-2, MMP-9. The results show that CTX inhibited the secretion of VEGF and MMP-2 in macrophage supernatants.

Interestingly, MMPs, especially MMP-2 and MMP-9, activate and release VEGF that is obtained by the extracellular matrix, allowing the migration of EC. In addition, the TNF-α is secreted by macrophages associated with tumour angiogenesis^33,37^. In our data, we observed that there was marked inhibition of TNF-α secretion in the supernatants obtained from macrophages pretreated with the toxin. This is in accordance to what was observed in macrophages culture treated with CTX^16^. It is important to remember that the decrease of the secretion of the angiogenic mediators in the supernatants is not a consequence of the loss of macrophages viability.

Additionally, these substances may also be regulated by lipid mediators such as LXA_4_, and its stable analogue, 15-epi-LXA_4_, described as potent antitumour and antiangiogenic factors^26,52–54^. It has been well-demonstrated by our group that CTX stimulates the secretion of these lipid mediators by macrophages, both *in vitro* assay^16,55^ as *in vivo* study^14^. Furthermore, it was shown that blocking the FPRs, ligands of these mediators prevent the release of these lipid mediators by macrophage pretreated with CTX^14,16^. Based on these facts, we investigated the possible participation of the FPR in the inhibitory activity of CTX on the effects of macrophages in the event of neovascularization. Indeed, our results show that the FPRs may contribute to this effect, since the Boc-2, a selective FPRs antagonist, blocked the inhibitory activity of macrophages treated with CTX on proliferation and cell adhesion assays. Also, pre-treatment with Boc-2 blocked the inhibitory effect of supernatants obtained on differentiated macrophages from human THP-1 cells on tube formation by human HUVEC cells. It is important to note that THP-1 cells express FPRs^56,57^. Therefore, our data demonstrate, for the first time, that FPRs are essential for the immunomodulatory actions of CTX also in human cells. Both Lipoxins and their stable analogues exert biological effects, such as antiangiogenic property, by binding to FPRs, inhibiting the secretion of some mediators, such as TNF-α, VEGF and MMPs, leading to inhibition of the cell proliferation and tumour growth^52,58–61^. Thus, we can suggest that the inhibitory action on the secretion of pro-angiogenic mediators may be due to the release of LXA_4_/15-epi-LXA_4_ by macrophages treated with CTX, demonstrated in previous studies^14,16,55^.

### Concluding remarks

**Figure 9:**
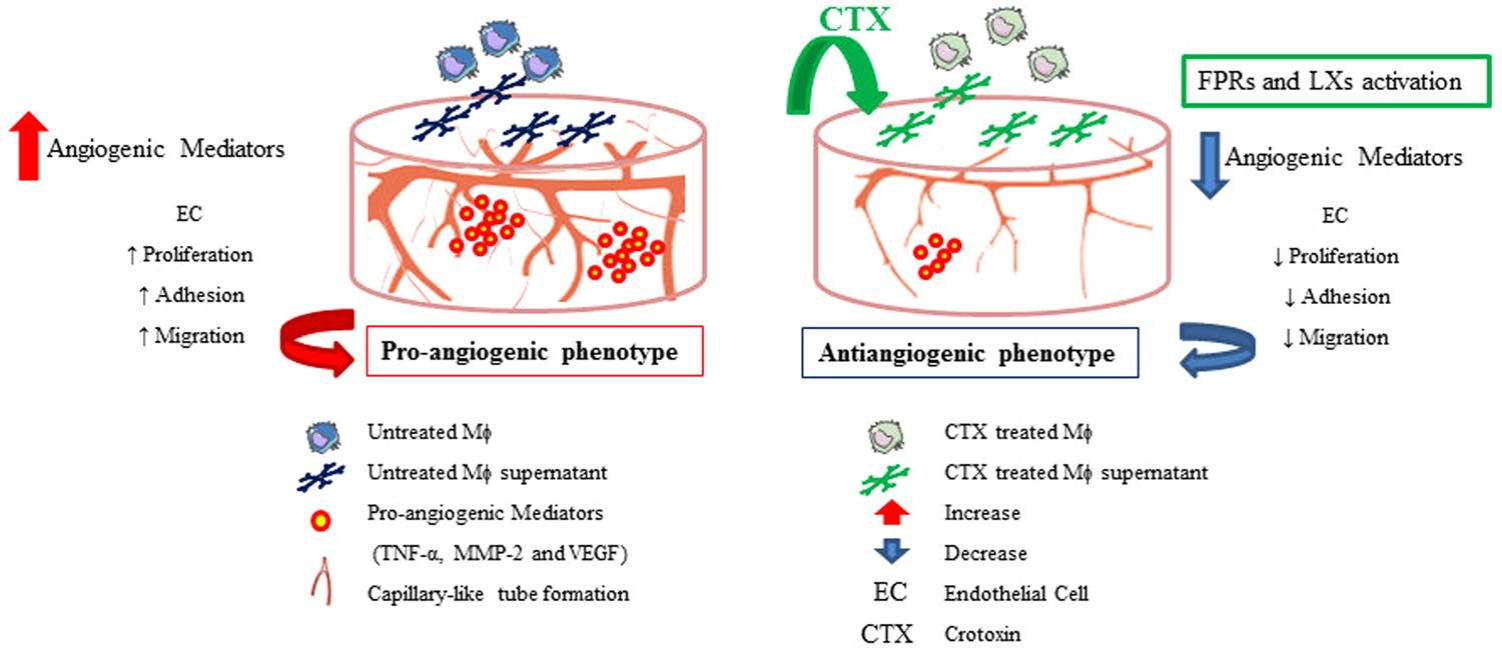
Scheme proposed for macrophage reprogramming for an antiangiogenic phenotype induced by CTX *in vitro*. CTX, with the participation of the FPRs, leads to a decrease the secretion of angiogenic mediators by macrophages, inhibiting the events involved with neovascularization, such as adhesion, proliferation and migration of EC and, consequently, the reduction of the capillary-like tube formation. Both the blockade these FPRs and CTX-induced lipoxins may contribute to the antiangiogenic activity of macrophages, since these lipid mediators can lead, for example, the inhibition of some angiogenic mediators. Thus, the antiangiogenic action of macrophages induced by CTX may be crucial to the antitumour effect described for this toxin in different *in vivo* studies.

As summarized in Fig. 9, CTX-treated macrophages or supernatants of cultured CTX-treated macrophages inhibited EC proliferation, adhesion, and migration that are closely associated with angiogenesis and neovascularization. The CTX antiangiogenic effect resulted of a decrease in the secretion of the pro-angiogenic factors MMP-2, TNF-α and VEGF. This antiangiogenic effect is blocked by the FPRs antagonist, suggesting that these receptors, as well as LXA_4_ and 15-epi-LXA_4_, participate in the antiangiogenic activity of macrophages treated with this toxin. Therefore, we can suggest that CTX treatment reprogrammed macrophages from usual proangiogenic to an antiangiogenic phenotype. This phenotypic reprogramming of macrophages may contribute significantly to control the reduction of the angiogenic process associated with the antitumour activity of the CTX previously reported in experimental studies^7–9^, for review^10,14^. These findings contribute to elucidate the mechanisms involved in the antitumor properties of the CTX reported in phase I clinical trials with patients with solid tumours refractory to conventional therapy^7^ and are under patent request analysis (Public Patent US 2013/0129706 A1). Therefore, considering the phases involving the clinical studies, the data obtained in the present study contribute significantly to the advancement of these analyzes, since it expands the discussion about the crucial participation of the immunomodulatory capacity of CTX on macrophages in the microenvironment tumor. Furthermore, the use of CTX to understand the mechanisms involved in triggering and controlling the phenotypic reprogramming of macrophages opens up prospects for immunotherapy, an important alternative treatment for the overcoming of different inflammatory diseases, including tumor development.

## Materials and Methods

### Animals

Male Wistar rats weighing 160–180 g were used in this study. All procedures were performed following the guidelines for animal experimentation and the Ethical Committee for the Use of Animals of the Butantan Institute approved the protocol (CEUAIB, protocol number 1052/13).

### CTX

As described in other studies^14,16,55,62^, crude venom solution was subjected to anion-exchange chromatography as previously described by Rangel-Santos *et al*.^63^, using a Mono-Q HR 5/5 column in an FPLC system (Pharmacia, Uppsala, Sweden). The fractions (1 mL/min) were eluted using a linear gradient of NaCl (0–1 mol/L in 50 mmol/L Tris-HCl, pH 7.0). Three peaks (p1, p2 and p3) were obtained: p2 corresponded to the pure CTX fraction (about 60% of the crude venom); peaks 1 and 3 included the other CdtV toxins. Before pooling, the fractions containing CTX were tested for homogeneity by non-reducing sodium dodecyl sulphate-polyacrylamide gel electrophoresis (12.5%)^64^ and the phospholipase A_2_ activity was assessed by a colorimetric assay using a synthetic chromogenic substrate^65^.

### Peritoneal cell preparation

As described by Costa and colleagues^16^, the animals were euthanized in a CO_2_ chamber, and the peritoneal cavity was opened and washed with 10 mL of cold phosphate-buffered saline (PBS), pH 7.4. After a gentle massage of the abdominal wall, the peritoneal fluid containing the resident macrophages was collected. The number of total peritoneal cells was determined using a Neubauer’s chamber. Samples from individual animals were used for all measurements.

### THP-1-macrophage differentiated preparation

THP-1 cells (1 × 10^5^/mL) were cultured in RPMI medium containing fetal bovine serum (FBS) at 10% and L-glutamine 1% in an oven at 37 °C and 5% CO_2_ for 120 hours. The subculture of the cells was performed every 2 days. Afterwards, the cells were collected, centrifuged and re-suspended in the fresh culture medium. To obtain THP-1-macrophage differentiated, these cells (1 × 10^6^/well) in 6-well culture plates were incubated with PMA (100 nM), for 2 days. After this period, these cells were cultured in RPMI medium containing fetal bovine serum (FBS) at 10% and L-glutamine 1% in the oven at 37 °C and 5% CO_2_ for 72 hours. The cells were then used in the experimental assays.

### Endothelial cells culture

Were utilized two endothelial cell lineages (EC): Endothelial lineage of thymic cells derived from C57BL/6 mouse endothelioma thymus, established as endothelial by Williams and colleagues^66^, and are widely used^67–70^ and Human Umbilical Vein/Vascular Endothelium Cell lineage (HUVECC [HUVEC] (ATCC CRL1730™) from ATCC®. These cells (5 × 10^4^/mL in 10 mL) were cultured in RPMI medium containing fetal bovine serum (FBS) at 10% and L-glutamine 1% in an oven at 37 °C and 5% CO_2_ for 72 hours. After reaching the state of semi-confluence (80%), the cells were treated with trypsin/EDTA and used for the experiments. The subculture of the cells was performed every 2 days using 0.25% trypsin in PBS for 5 minutes in the oven at 37 °C. Afterwards, the cells were collected, centrifuged and re-suspended in the fresh culture medium.

### Pharmacological treatments

#### CTX treatment

**Figure 10:**
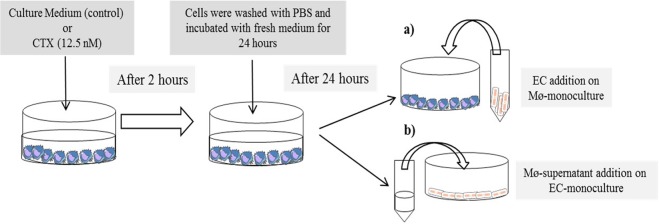
Experimental Procedure Scheme. Macrophages were obtained as described above and led to adhere (1 × 10^6^/ml) in 6-well culture plates for 1 hour at 37 °C and 5% CO_2_. Cells were then washed with PBS and incubated in the presence of CTX (12.5 nM, corresponding to 0.3 µg/mL in RPMI-1640 culture medium) for 2 h at 37 °C and 5% CO_2_. The dose of CTX used has marked effects on macrophage metabolism and function as reported in our previous studies 15,16,55,62. Afterwards, macrophages were washed with PBS and incubated in the presence of a fresh culture medium for 24 h at 37 °C and 5% CO_2_. (**a**) In order to perform the experiments of co-culture of macrophages with endothelial cells (EC), after 2 h of CTX treatment and 24 h of incubation in fresh medium, supernatants were collected and the macrophage monocultures were incubated in the presence of EC (5 × 10^5^ cells/well) for 24 h at 37 °C and 5% CO_2_. (**b**) To investigate the indirect interaction between EC with secreted mediators (cell-cell no-contact), supernatants collected from macrophage monocultures treated with CTX for 2 h and incubated in fresh medium for 24 h were centrifuged to remove cellular debris and then incubated in cultured EC, at different periods of time.

Macrophages were obtained as described above and led to adhere (1 × 10^6^/ml) in 6-well culture plates for 1 hour at 37 °C and 5% CO_2_. Cells were then washed with PBS and incubated in the presence of CTX (12.5 nM, corresponding to 0.3 µg/mL in RPMI-1640 culture medium) for 2 h at 37 °C and 5% CO_2_. The dose of CTX used has marked effects on macrophage metabolism and function as reported in our previous studies^15,16,55,62^. Afterwards, macrophages were washed with PBS and incubated in the presence of a fresh culture medium for 24 h at 37 °C and 5% CO_2_. In order to perform the experiments of co-culture of macrophages with endothelial cells (EC), after 2 h of CTX treatment and 24 h of incubation in fresh medium, supernatants were collected and the macrophage monocultures were incubated in the presence of EC (5 × 10^5^ cells/well) for 24 h at 37 °C and 5% CO_2_. To investigate the indirect interaction between EC with secreted mediators (cell-cell no-contact), supernatants collected from macrophage monocultures treated with CTX for 2 h and incubated in fresh medium for 24 h were centrifuged to remove cellular debris and then incubated in cultured EC, at different periods of time. The experimental design of the treatment is summarized below in the Fig. 10*. Experimental Procedure Scheme*. The protocols used were based on previous studies^16,27,50,71–73^.

#### Incubation with Boc-2, an antagonist of the formyl peptide receptor (FPR)

Macrophages were left to adhere for 1 h at 37 °C and 5% CO_2_. The cells were then washed and incubated in 75 cm^3^ culture bottles in the presence of 100 µM Boc-2, a selective FPR antagonist, for 15 minutes (for rat macrophage)^16^ or 30 min (for THP-1-macrophage differentiated) at 37 °C. After that, the macrophages were washed with PBS and incubated with CTX (12.5 nM) for 2 h at 37 °C and 5% CO_2_. After this period, monocultures were washed and incubated in fresh culture medium for 24 h at 37 °C and 5% CO_2_. The supernatants were then collected to evaluate the involvement of FPRs in the CTX effects.

#### Cell proliferation measurement

EC (5 × 10^5^ cells/well) were left to adhere in 6-well plates for 2 h at 37 °C and 5% CO_2_. After this period, the wells were washed, and the supernatants from macrophages treated with CTX were then added. After 24 hours of culture, supernatants were removed, and the EC were treated with trypsin, re-suspended in PBS containing blue dye Trypan at 1% and the total number of cells counted in a Neubauer chamber, using a light microscope as described by Hotchkiss *et al*.^74^. Blue exclusion in viable cells determined cell viability.

#### Adhesion to natural ligands assay

A suspension of EC (1 × 10^6^/mL) was incubated in the absence or presence of macrophage supernatants obtained from macrophages cultured for 24 h at 5% CO_2_ and 37 °C. Next, the cells were washed and suspended in 1 mL serum-free culture medium. A cell suspension (100 µl/well) was added to the plates (Maxsorp®, Nunc) containing 96 wells, overnight treated with different matrix components type I collagen (from rat tail- Gibco®), 10 µg/well^75^; fibronectin (from human plasma- Gibco®), 3 μg/well^75^ and laminin (natural mouse laminin isolated from the Engelbreth-Holm-Swarm sarcoma- Invitrogen®), 10 µg/well^76^ at 8 °C. A negative control was included by treating wells only with albumin (1% BSA). After 24 h, the plates were washed with PBS, and 100 µL of 1% BSA were added to each well to block nonspecific sites. After 1 hour adherence, cells not adhered were removed from the wells by two consecutive washes with PBS. The adhered cells were incubated with 100 µL serum-free culture medium and 30 µL of MTT (3- (4,5dimethylthiazol-2-yl) -2,5-diphenyl tetrasodium bromide - Amresco® Solon, USA) at 5 mg/mL PBS, for 3 hours. The dye MTT has a tretazol yellow color (oxidized form) that is converted to an insoluble formazan (reduced form) dark blue compound in viable cells. This change in colors is induced by mitochondrial dehydrogenase activities. Afterwards, 100 µL of 10% SDS dissolved in distilled water (containing 0.01 M HCl) were added to each well. The plates were kept for 18 hours at 37 °C and 5% CO_2_. After this period, the precipitate was solubilized and quantified using spectrophotometry at 595 nm in ELISA reader (Multiskan EX Labsystem®, Molecular Device, California, USA). The standard curves were prepared to start with 10^6^ cells/mL using number of cells corresponding to 100, 80, 65, 50, 35, and 20 μL of the suspension. Each experiment was performed eight times. The number of adhered cells was calculated using the GraphPad INSTAT program Software V2.01.

#### *In vitro* wound healing assay

This assay was used to evaluate the movement of cells in the empty field created by an interruption in the EC monolayer after 24 h in culture^77^. To obtain a confluent cell monolayer, EC were re-suspended in RPMI-1640 culture medium containing 10% FBS. This cell suspension (5.5 × 10^5^ cells/1 mL/well) was adhered to 24 well plates containing glass coverslips previously coated with type I collagen (10 µg/100 µL) for 1 h at 37 °C and 5% CO_2_. Afterwards, an interruption was made using a sterile pipette tip (200 μL) to create a cell-free area. To obtain the T0, after the completion of the disruption of the monolayer, some coverslips were fixed and stained with Rosenfeld dye^78^. The other coverslips were washed with PBS to remove the suspended cells and then incubated in the presence of the supernatants obtained from cultured macrophages. Simultaneously, the second protocol was performed where EC adhered to the wells in the presence of macrophages pre-treated or not with CTX. Under this condition, the ratio of the cells was 1:1 (5 × 10^5^ cells of each cell type) and 1 × 10^6^ cells were used to obtain a confluent monolayer. After the interruption of the monolayer, coverslips were incubated in the presence of RPMI-1640 culture medium. In both protocols, the migration assay was performed for 24 h at 37 °C and 5% CO_2_. After this period, supernatants were removed, and the coverslips were fixed and stained with Rosenfeld dye removed from the culture plate wells and mounted on slides with the aid of Entellan® Merck Millipore, Darmstadt, Germany). At least five different fields per coverslip were photographed using a Leica DFC 420 Olympus BX51 microscope and the Image-Pro Plus 5.1 software. For counting the migrating cells in the field induced by the probe, the images were inserted into the rules on the left and right edges of the field, based on the image obtained in the T0 coverslip. After insertion of dashed lines, the count of migrating cells in the field was performed^77,79^.

#### Wound healing and Cell Migration assays in Time-Lapse Video Microscopy

For the wound healing assay, EC (2.5 × 10^5^ cells/well) were adhered to TTP® 24-well plates coated with type I collagen (10 µg/100 µL) and incubated overnight at 37 °C and 5% CO_2_. After this period, an interruption was created in monolayer as described above. The wells were then washed and incubated in the presence of supernatants obtained from macrophage cultures and incubated in the 2200 cell analyzer system for 24 h at 37 °C and 5% CO_2_. The images were obtained in 10x objective and were used to determine the distance between the margins after the different treatments. In addition, Time-Lapse Video Microscopy and Analysis of Cell Migration were evaluated. For this, TTP® 24-well plates coated with type I collagen (10 µg/100 µL) for 30 min at 37 °C. Then the plates were washed 3 times with PBS and 1 × 10^3^ cells/well were plated and incubated in RPMI for 24 hours. After this period, the EC were incubated in the presence of supernatants from CTX-macrophages. Then, the plate was coupled to the equipment InCell Analyzer 2200 GE in the 10x air objective lens and 8 fields/well (6 wells for each treatment) were recorded every 5 min for at least 24 h. Image acquisition was performed with Analyzer 2200 version 1.6.3. Cell nuclei were tracked by the ImageJ plugin manual tracking. Thus, the velocity of the cells was analyzed and the net distances per hour were calculated and also summed up to determine the total path lengths of the cells, as described by Hauff *et al*.^80^.

#### Capillary-like tube formation on matrigel-3D matrix

Matrigel (Matrigel BD Biosciences®, USA) was diluted in serum-free medium at 0.9 mg/mL, and 60 μL/well of Matrigel were added to 96-well plates. The plates were maintained for 40 min at 37 °C and 5% CO_2_ for the polymerization of the gel. EC cells (2.5 × 10^4^) were suspended in 50 µL RPMI-1640 culture medium or 50 µL of supernatants obtained from macrophage monolayer treated or not with CTX. The cells were then added on matrigel and incubated for 2 h at 37 °C and 5% CO_2_. After this period, images were captured, and the number of structures was quantified by counting all observed branches, at least six random fields, as previously described^71,73^. The images were captured a Nikon Eclipse® TS100 microscope, equipped with a DS-Fi2 camera and 10x objective using the Nis-Elements software, Dexter, MI, USA).

#### Determination of the angiogenic factors

The angiogenic factors present in the supernatants of the cell cultures were quantified using ELISA. Briefly, ELISA plates (Immuno Maxisorp®; Nunc, Thermo Fisher Scientific, MA, USA) were coated with mouse anti-rat monoclonal or polyclonal antibodies against MMP-2 e MMP-9, VEGF and TNF-α (R&D Systems®, Minneapolis, MN and Abcam® Cambridge, UK). The plates were incubated overnight at room temperature and blocked for 1 h at room temperature on a shaker before adding the samples and standards. Biotinylated secondary antibodies were added 2 h before the incubation, and peroxidase-conjugated streptavidin was added 20 min before incubation. Tetramethyl benzidine (TMB), a peroxidase substrate, was added, and the plates were incubated in the dark for 20 min for color development. The reaction was stopped with 2 N sulphuric acid, and the plate colors were read using dual wavelengths (465 and 590 nm) on a microplate reader (Spectra Max 190, Molecular Devices, California, USA). The mediator concentrations were determined by comparison with a standard curve prepared using the recombinant murine mediators (R&D Systems®, Minneapolis, USA and Abcam®, Cambridge, UK). Concentrations of the mediators were expressed in picograms per 10^6^ macrophages.

### Statistical analysis

Statistical analyses of the differences between the groups were performed according to Glantz^81^ using GraphPad InStat software version 3.01 (GraphPad Software Inc., San Diego, CA, USA). One-way analysis of variance followed by Tukey’s test was used for multiple comparisons (all pairs of groups) of the values from the assays using Boc-2. To analyze the results from other assays, one-way analysis of variance followed by Bonferroni’s test was used for multiple comparisons against a single control or by an unpaired Student t-test or Mann Whitney test to compare two groups. Differences with p < 0.05 were considered statistically significant. The results are presented as mean values ± standard errors of means.

## Supplementary information

Effect of supernatant from untreated-macrophages on EC migration evaluated in time-lapse assay

Effect of supernatant from CTX-macrophages on EC migration evaluated in time-lapse assay

Effect of supernatant from untreated-macrophages on cytoskeleton dynamic EC during migration, evaluated in time-lapse assay

Effect of supernatant from CTX-macrophages on cytoskeleton dynamic EC during migration, evaluated in time-lapse assay

Legends of the S1 FIGURE and VIDEOS

Dataset 1
