# Supplementary material for: Crotoxin promotes macrophage reprogramming towards an antiangiogenic phenotype
Source: Sci Rep. 2019 Mar 12;9:4281. doi: 10.1038/s41598-019-40903-0 (PMC6414609; doi:10.1038/s41598-019-40903-0)
Supplement: Supplementary file 5 — Legends of the S1 FIGURE and VIDEOS [file 41598_2019_40903_MOESM5_ESM.docx]

**Crotoxin promotes macrophage reprogramming towards an antiangiogenic phenotype**

Luciana de Araújo Pimenta^a,b^ ^&^, Maíra Estanislau S. de Almeida^a,c &^, Marisa Langeani Bretones^a^, Maria Cristina Cirillo^a^, Rui Curi^d,e^, Sandra Coccuzzo Sampaio^a,b^*

*^a^Laboratory of Pathophysiology, Butantan Institute, Av. Vital Brazil, 1500, 05503-900, SP, Brazil*

*^b^Department of Pharmacology,*

*^c^Department of Cell and Developmental Biology,*

*^d^Department of Physiology and Biophysics, Institute of Biomedical Sciences, University of São Paulo, Av. Prof. Lineu Prestes, 1524, 05508-900, SP, Brazil*

*^e^Interdisciplinar Post-Graduate Program in Health Sciences, Cruzeiro do Sul University, 868 Galvão Bueno, 01506-000, São Paulo, Brazil*

**Running title**: Crotoxin promotes antiangiogenic macrophage reprograming

*Corresponding author: Dr. Sandra Coccuzzo Sampaio, Laboratory of Pathophysiology, Butantan Institute, Av. Vital Brazil, 1500, 05503-900, São Paulo, Brazil, Phone: (55) (11) 2627-9562; Fax: (55) (11) 2627-9581; e-mail: sandra.coccuzzo@butantan.gov.br

^&^Both authors equally contributed to this article

**FIG. S1 OF THE MATERIAL SUPPLEMENTARY**


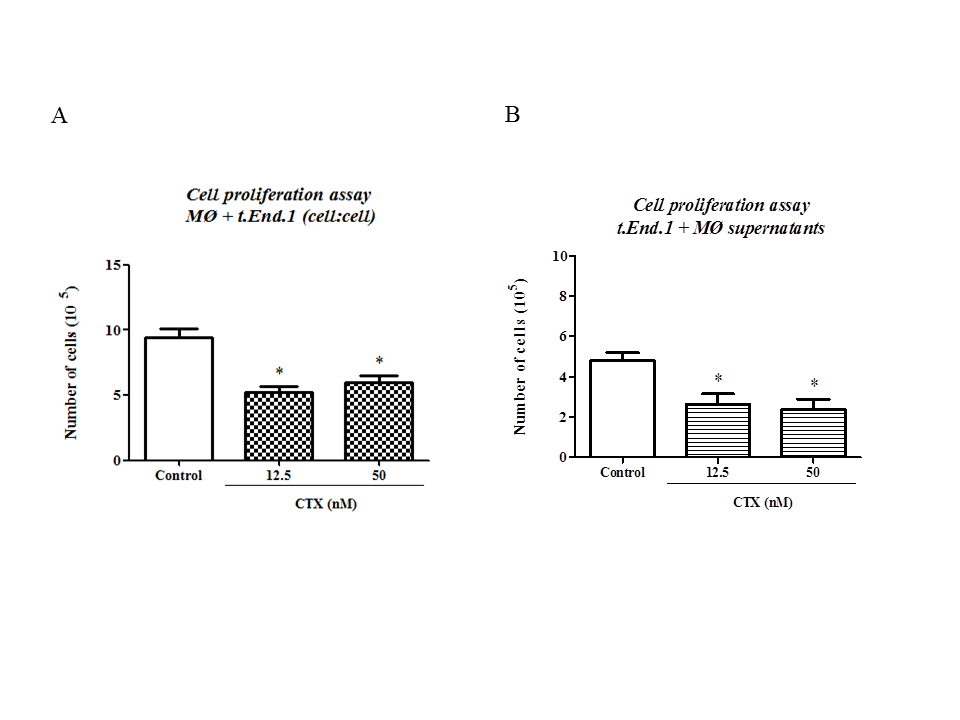


**Figure S1. Effect of co-cultures of macrophages pretreated with different concentrations of CTX on the EC proliferation**. In (A) resident macrophages were obtained from the rat peritoneal cavity, left to adhere (1x10^6^/well) in 6-well plates and incubated with CTX (12.5 nM or 50 nM) for 2 h. EC (5x10^5^ cells/well, in 24-well plates) were incubated in the presence of CTX treated or untreated macrophages for 24 hours at 37^o^C and 5% CO_2_. In (B) EC were also incubated in culture medium containing supernatants of CTX treated or untreated macrophages under similar conditions. For details of the experiments, please see Materials and Methods section. The results are expressed as number of cells and presented as mean ± s.e.m. of six samples per group of three distinct assays. *p <0.05, significantly different from mean values for groups to their respective controls (t.End.1+Untreated Mϕ or t.End.1+ Untreated Mϕ supernatant).

**LEGENDS OF THE VIDEOS – SUPPLEMENTARY MATERIAL**

**Video S1. Effect of supernatant from untreated-macrophages on EC migration evaluated in time-lapse assay**. The 24 well plates were coated with type I collagen (10 µg/mL) for 30 min at 37 °C. Then the plates were washed 3 times with PBS and 1x10^3^ cells/well were plated and incubated in RPMI for 24 hours. After this period, the EC were incubated in the presence of supernatants from untreated-macrophages. Then, the plate was coupled to the equipment InCell Analyzer 2200 GE in the 10x objective, for 24 h.

**Video S2. Effect of supernatant from CTX-macrophages on EC migration evaluated in time-lapse assay**. The 24 well plates were coated with type I collagen (10 µg/mL) for 30 min at 37 °C. Then the plates were washed 3 times with PBS and 1x10^3^ cells/well were plated and incubated in RPMI for 24 hours. After this period, the EC were incubated in the presence of supernatants from CTX-macrophages. Then, the plate was coupled to the equipment InCell Analyzer 2200 GE in the 10x objective, for 24 h.

**Video S3. Effect of supernatant from untreated-macrophages on cytoskeleton dynamic EC during migration, evaluated in time-lapse assay**. The 24 well plates were coated with type I collagen (10 µg/mL) for 30 min at 37 °C. Then the plates were washed 3 times with PBS and 1x10^3^ cells/well were plated and incubated in RPMI for 24 hours. After this period, the EC were incubated in the presence of supernatants from untreated-macrophages. Then, the plate was coupled to the equipment InCell Analyzer 2200 GE in the 60x objective, for 24 h.

**Video S4. Effect of supernatant from CTX-macrophages on cytoskeleton dynamic EC during migration, evaluated in time-lapse assay**. The 24 well plates were coated with type I collagen (10 µg/mL) for 30 min at 37 °C. Then the plates were washed 3 times with PBS and 1x10^3^ cells/well were plated and incubated in RPMI for 24 hours. After this period, the EC were incubated in the presence of supernatants from CTX-macrophages. Then, the plate was coupled to the equipment InCell Analyzer 2200 GE in the 60x objective, for 24 h.
